# External root resorption of second molars due to impacted third molars

**DOI:** 10.4317/jced.62080

**Published:** 2024-12-01

**Authors:** Shehab Ahmed Hamad

**Affiliations:** 1 BDS, MSc, MOMSRCPS, MFDTRCSED, FIBMS, FFDRCSI(OSOM), FDSRCS, FDSRCPS, FICD. Professor of maxillofacial surgery. Kurdistan Higher Council of Medical Specialties. Ziraah Square, Erbil, Iraq

## Abstract

**Background:**

The current study attempts to assess the impact of third molar impaction on external root resorption (ERR) of the adjacent second molars. We aimed to determine the prevalence, severity, and associated factors with ERR in a sample of panoramic radiographs.

**Material and Methods:**

A retrospective cross-sectional study was carried out at teaching hospital. We reviewed panoramic radiographs from September 2021 to September 2024, selecting images with second and third molars in patients over 16 years old and impacted third molars. ERR was analyzed with respect to patient age and sex. The presence of symptoms, site and severity of root resorption, relationship to angulation, and depth of impaction were also recorded.

**Results:**

A total of 750 panoramic radiographs were examined, and ERR was observed in 32 cases (4.30%). ERR was slightly more common in males (62.5%) than in females (37.5%), but the difference was not statistically significant. The maximum number of ERR cases was found in the age group of 16-25 years (68.7%). Most classification of ERR was mild (71.8%). Mesioangular impacted third molars were associated with 65.6% of resorption cases and position C third molars were associated with 90.6% of resorption cases. The middle third of the root was the most common site of ERR (59.3%).

**Conclusions:**

ERR of second molars was relatively more common among patients with impacted third molars. Associations were significant for angulation (mesioangular), depth of impaction, and ERR. Hence, a question arises in assessing the amount of risk associated with its impaction in panoramic radiographic imaging.

** Key words:**Impacted, digital panoramic radiography, external root resorption, second molar, third molar.

## Introduction

An impacted third molar may cause pericoronitis, caries of the second and third molars, odontogenic cysts, and crowding of anterior teeth. It may also cause periodontal destruction of the adjacent second molar and even ERR (1). Wang *et al*. has shown that the prevalence of ERR in second molars can exceed 20% in samples that include both maxillary and mandibular third molars, increasing to 50% when only mandibular third molars are involved (2).

The degree of resorption depends on various factors, including the angle of impaction, the proximity of the third molar to the second molar, and the age of the patient. Third molars that are mesio-angulated and deeply impacted have been recognized as significant risk factors for root resorption in both maxillary and mandibular second molars. Additionally, individuals over the age of 25 exhibit an elevated risk of experiencing root resorption in their second molars. In the case of maxillary second molars, root resorption predominantly takes place in the apical third, whereas for mandibular second molars, it is most commonly observed in the cervical third (3).

The ERR of second molars has been observed at the location where the impacted tooth makes contact, suggesting that the pressure from the impacted tooth may play a role in the resorption process (4). The occurrence of awake bruxism and increased activity of the masticatory muscles appears to be associated with the presence of ERR in the second molars that are adjacent to impacted mandibular third molars (5). Although the exact mechanism of this resorption remains unclear. Some research indicates that this phenomenon bears resemblance to the resorption of primary teeth by emerging permanent teeth. In both instances, the resorption is influenced by mechanical forces and cellular processes that contribute to the degradation of root structure (6). Resorption of roots occur when cementoblasts from the tooth’s outer layer are removed, the root surface is exposed. This causes neighboring osteoclasts to become activated, which in turn promotes root resorption (7).

Irreversible damage is a significant concern of ERR. While mild ERR may result in a shortened or blunt root structure, severe ERR can lead to dental pulp diseases and various lesions, compromising the stability and chewing functions of the teeth, ultimately necessitating the extraction of affected teeth. Even mild to moderate cases of ERR can result in a reduction of the periodontal tissues surrounding the adjacent third molar. Nevertheless, research indicates that there are typically no symptoms associated with mild or moderate ERR lesions that have not progressed to the dental pulp, making them difficult to detect through clinical examination alone; thus, imaging techniques are essential for accurate diagnosis (8)

The objective of the present study was assess the severity and location of external root resorption of second molars and the association with the age and sex of the patients and also the depth and angulation of impacted third molars.

## Material and Methods

-Design of the Study and Selection of the Sample

This is a retrospective cross-sectional. Ethical approval was granted by the Institutional Review Board at Kurdistan Higher Council of medical Specialties (Protocol Number:102). Panoramic radiographs and related clinical examination records for patients referred for various dental procedures over a 3-year period from September 2021 to September 2024 were reviewed.

Panoramic radiographs taken as a diagnostic aid in the university’s dental clinics were collected. For this study we selected radiographs that clearly displayed an area of interest which are mainly second and third molars in mandible and maxilla. Patients whose age was more than 16 years old with one impacted third molar located next to a second molar were involved. Inclusion criteria were fulfilled if there existed evidence on radiograph which indicated that either a fully or almost fully formed third molar (at least two-thirds root formation) had no major caries lesion present nor evidence of cysts, tumors or other pathologies connected with these teeth. Radiographs with artifacts or having high density materials obstructing areas of interest were excluded also.

-Radiographic Analysis

For the panoramic radiographs, two oral and maxillofacial surgeons who were unaware of patients’ clinical details analyzed them using Panoramic Dental Application (PDApp). However, depth and angulation of the impacted third molars were assessed according to Pell and Gregory’s classification for depth and Winter’s classification for angulation.

-Resorption Evaluation

Using the criteria of Ericson *et al*. (9), in this case, ERR of the second molar suggests a loss of substance at the distal surface of the second molar because of direct contact with the impacted third molar. ERR may be sub-classified as slight, moderate or severe:

• Slight: Less than half the thickness of dentin is involved.

• Moderate: At least half of the thickness of dentin is involved.

• Deep: Extending into the pulp chamber.

The level of ERR on the second molar root was recorded as cervical, middle, or apical third.

-Clinical Examination

Apart from radiographic examination, clinical examination charts were screened for any complaints associated with second molars and impacted third molars.

-Data Collection and Statistical Analysis

Demographic data also included the age and gender of the patients. The ages of the patients were grouped into three categories: 16-25 years, 26-35 years, and above 35 years. The data collected was analyzed using SPSS version [insert version] (SPSS Inc., Chicago, IL, USA). The incidence and severity of ERR were related to the depth and angulation of impacted third molars, as well as patient age and sex. Intra- and inter-rater reliability was calculated with Cohen’s kappa coefficient. Associations between categorical variables were tested with a Z-test for proportions and Chi-square test at a significance level of *p* < 0.05.

## Results

The details of patient characteristics, symptoms presentation, severity and location of external root resorption, and the vertical position and angulation of impacted third molars are presented in  Table 1.

**Figure 1:**
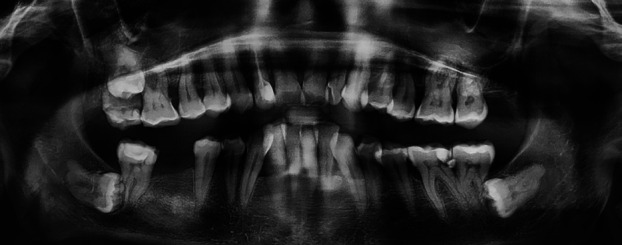
Severe resorption of maxillary right and mandibular right and left second molars by impacted third molars.

**Figure 2:**
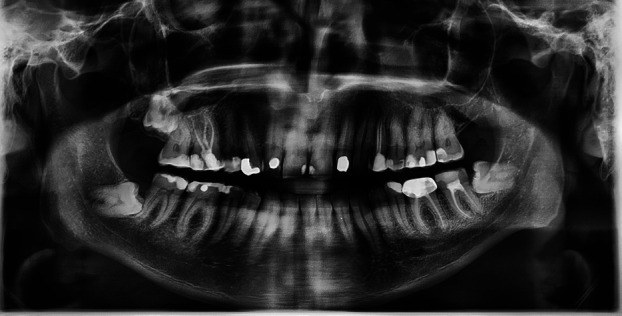
Mild root resorption associated with horizontal, position C impacted third molar.

**Figure 3:**
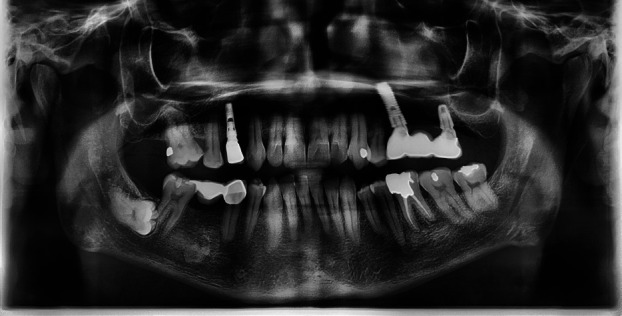
Severe root resorption associated with horizontal, position C impacted third molar.

Inclusion criteria were met and 750 panoramic radiographs were evaluated, with gender distribution of 420 males (56%) and 330 females (44%). Thirty-two patients had second molar ERR (30 mandibular and 2 maxillary) caused by impacted third molars (Figs. 1,2,3). This is equal to a percentage of 4.27% among the samples (0.26% of the maxillary and 4% of mandibular molars). External root resorption was more common in men than women with ERR being higher in males (20 cases, 62.5%) than females (12 cases, 37.5%), however, it was not statistically significant ( *p* > 0.05).

External root resorption mostly occurred within the age group of 16-25 years with a total of 18 cases (68.75%), followed by age group 26-35 years having 7 cases (21.87%), then from 36-45 years had 6 cases (18.75%), and lastly one case (3.12%) in the 46-55 years age group. This age pattern of ERR was statistically significant; *P* < 0.01. Twenty-six cases (81.25%) of ERR patients had no symptoms hence asymptomatic while other 6 (18.75%) experienced pain and tenderness on the affected second molar; this difference was significant; *p*<0.01.

Based on severity, mild accounted for the majority with 23 (71.87%) cases, moderate resorption seen in 6 cases (8.75%), while 3 cases (9.37%) showed severe status. The severity distribution was significantly different; *p*< 0.001.

The majority of cases in relation to ERR of second molars with depth impacted third molars were found at position C (90.62%, 29 cases), followed by position B (9.37%, 3 cases). Position A was not related to any ERR cases, and this distribution across various positions was statistically significant; *p*< 0.001.

External root resorption was associated mostly with mesioangular impactions (21 cases, 65.62%), followed by horizontal impactions (11 cases, 34.37%), with no other angulations noted. Mesioangular angulation correlated significantly with ERR (*p* < 0.05). Most common location of ERR was the middle third of the root (19cases, 59.37%), followed by cervical third (8cases, 25%) and apical third (5 cases, 15.62%). This pattern was highly significant; *p*<0.01.

## Discussion

This study evaluated 750 panoramic radiographs and clinical records and found that 32 patients (4.27%) exhibited ERR of the second molar caused by impacted third molars. CBCT based studies reported higher incidence of ERR than the present study. Lacerda-Santos *et al*. (10) found an incidence of ERR of 47.7% and Sakhdari *et al*. (11) reported the frequency of second molar ERR was 33.4% in the mandible and 14% in the maxilla.

The gender distribution in our study showed a higher prevalence in males (62.5%) compared to females (37.5%), though this difference was not statistically significant. Certain studies have indicated that male subjects exhibit a greater prevalence of second molar external root resorption (ERR) linked to the presence of impacted third molars, which may be attributed to variations in jaw or tooth size. Researchers attribute this phenomenon to the effects of sex hormones. Conversely, other studies (12,13) suggest that gender does not serve as a risk factor for second molar ERR, aligning with our findings.

Our results indicate that ERR was most common in the 16-25 years age group (68.75%). This finding is consistent with the literature, which often highlights that younger patients are at higher risk due to ongoing root development. For instance, a study by Oenning *et al*. (13) observed a higher incidence of ERR in younger patients, attributing this to the proximity of the impacted third molar and the active root formation in this age group.

Regarding symptomatic presentation, 81.25% of our patients were asymptomatic, which is in line with other research indicating that ERR is frequently asymptomatic. A study by Qu *et al*. (8) similarly reported that a large proportion of patients with ERR did not present symptoms, emphasizing the silent nature of many cases. The lack of significant differences in symptom presentation between genders further corroborates findings from other studies, which have also failed to establish a clear link between gender and symptomatic presentation of ERR.

Our severity distribution showed that mild ERR was most common (71.87%), with moderate (18.75%) and severe (9.37%) cases less frequent. These results are consistent with the findings of Li *et al*. (14), who reported that mild and moderate ERR were more prevalent compared to severe cases in their study population, in both the maxilla and mandible.

The depth of impaction analysis revealed that ERR was predominantly associated with third molars in position C (90.62%), with a statistically significant distribution. This finding aligns with previous research indicating that deeper impactions are more likely to cause ERR. For example, a study by Smailienė *et al*. (15) found a significant association between the depth of impaction and the occurrence of ERR, supporting the relevance of impaction depth in ERR development.

Mesioangular impactions were significantly correlated with ERR (65.62%), compared to horizontal impactions (34.37%). This is consistent with the findings of Keskin Tunç and Koc (16) and Akkitap and Gumru (17), who reported that mesioangular impactions are more commonly associated with ERR due to their positioning relative to the adjacent molar. The statistical significance of this correlation reinforces the importance of impaction angulation in predicting ERR.

Finally, the location of ERR was predominantly in the middle third of the root (59.37%), with a significant distribution. This pattern is consistent with previous studies, such as that by Schriber *et al*. (18), which found that The cervical third (28.6%) showed a significantly lower risk percentage of ERR compared to the apical (73.7%) and middle thirds (60.6%) of the root. The study attributed this to the anatomical and physiological factors influencing resorption at this site. Further more, Lacerda-Santos (10) found a statistically significant association between ERR location and severity; the cervical third was the most affected by mild ERR and the middle third was the most affected by severe ERR.

The limitation of this study is the use of panoramic radiograph for detecting the ERR which may underestimate the presence and severity of resorption. Several studies indicated a considerable level of concordance between panoramic radiograph and CBCT in detecting ERR; however, this is primarily attributed to the significant number of second molars identified as lacking ERR. For instance, in the investigation conducted by Oenning *et al*. (19), the agreement regarding the presence of ERR was observed at 4.3%, in contrast to 76% for the absence of ERR.

## Conclusions

In the current study, 4.27% of the patients developed ERR of second molars concerning the impact of third molars; a majority of the cases were noted among males between the age group of 16-25 years. A majority of the cases were presented as asymptomatic, and most of the cases were observed as the mild severity. The statistically significant associations were seen with the location of impactions and the locations of ERR. Therefore, the positions and angulations of third molars should be carefully accessed as part of any management plan for potential resorptive problems.

## Figures and Tables

**Table 1:** Patient characteristics, symptoms presentations, position of impacted third molars and severity and location of external root resorption.

Patient Characteristics	Number (n = 750)	ERR Cases (n = 32)	Percentage	P value
Male	420	20	62.5%	0.470
Female	330	12	37.5%	
16-25 years	200	18	56.25%	0.001
26-35 years	250	7	21.87%	
36-45 years	200	6	18.75%	
46-55 years	100	1	3.12%	
Symptoms of ERR				
Symptomless		26	81.25%	0.000
Pain/Tenderness		6	18.75%	
Severity of ERR				
Mild		23	71.87%	0.000
Moderate		6	18.75%	
Severe		3	9.37%	
Vertical position of impacted third molars				
Position C		29	90.62%	0.000
Position B		3	9.37%	
Position A		0	0%	
Angulation of impacted third molars				
Mesioangular		21	65.62%	1.00
Horizontal		11	34.37%	
Location of ERR				
Middle Third		19	59.37%	0.006
Cervical Third		8	25%	
Apical Third		5	15.62%	

## Data Availability

The datasets used and/or analyzed during the current study are available from the corresponding author.
